# Commentary: Conserved polymorphic sequences protect themselves for future challenges

**DOI:** 10.3389/fgene.2022.993944

**Published:** 2022-11-11

**Authors:** Roger L. Dawkins, Sally S. Lloyd

**Affiliations:** CY O’Connor ERADE Village Foundation, North Dandalup, WA, Australia

**Keywords:** recombination, haplotype, polymorphism, genomic architecture, major histocompability complex

## Introduction

**FIGURE 1:**
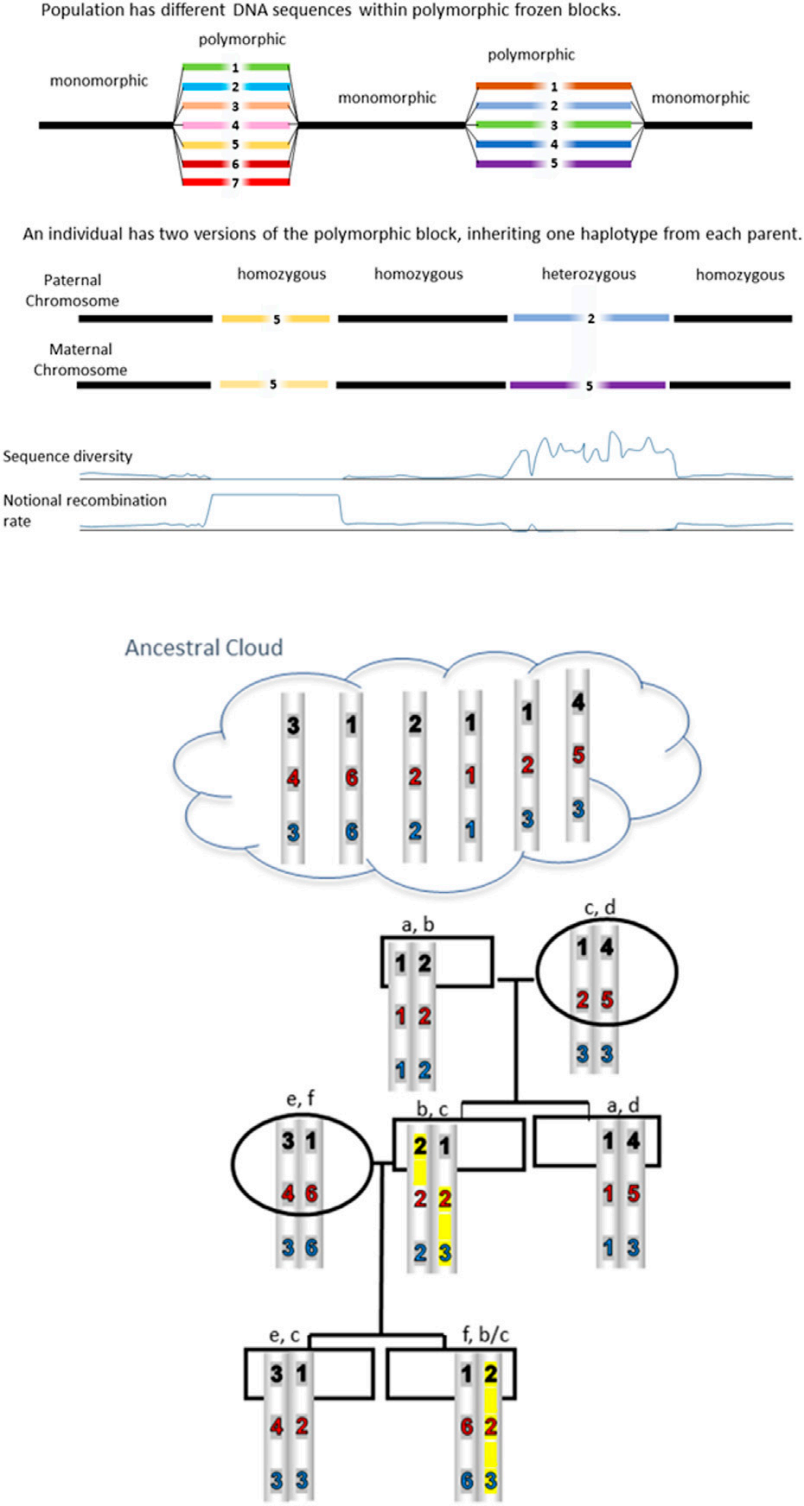
CPSs are inherited unchanged from distant ancestors. Blocks are conserved because sequence differences prevent recombination. Some reshuffling is possible when a homozygous block is present. An example is highlighted in yellow where the 123 haplotype has recombined with the 222 haplotype because it is homozygous at the second block thereby creating a 223 haplotype.

The Research Topic “Population genomic architecture: Conserved polymorphic sequences (CPSs), not linkage disequilibrium” addresses the discovery of distinct genomic regions or blocks which are highly polymorphic and therefore different between random individuals (Figure 1). Importantly, these differences are not due to recent mutation but have been conserved over hundreds of generations. So as avoid some of the confusion in the literature, we use mutation to describe an observed change in the sequence whereas polymorphism refers to a sequence difference which is inherited faithfully (Dawkins et al., 2013). This distinction is important but often overlooked. Polymorphisms which are “fixed” or “frozen” must somehow be protected from background mutation and recombination within the CPS. Just how this might happen has been of great interest and subject to much debate over several decades. For reviews see Steele (2014), Dawkins (2015), and Dawkins (2022).

## Conserved polymorphic sequences

The initial discoveries of Conserved Extended Haplotypes are presented in the chapter by Alper (2021). By the 1970s it was known that there could be extensive HLA haplotypes containing specific alleles of HLA-A and HLA-B which are nearly 1 Mb apart but inherited together, without recombination*.* It was soon appreciated that such subjects with HLA-A1 and HLA-B8 frequently had DR3 bringing the length of the A1, B8, DR3 or 8.1 haplotype to almost 2 Mb containing multiple genes, many of them duplicated. At the time there was much speculation as to how such “linkage disequilibrium” could be explained. Was there some form of *cis* interaction between the gene products? However, all became clear when the Alper group showed that individuals with 8.1 also have a specific complotype (SC01), unrelated to HLA, midway between HLA B and DR (Alper et al., 1982).

It was only a matter of time before methods, such as pulsed field electrophoresis (Tokunaga et al., 1989), allowed the direct demonstration of such an extensive DNA sequence. It transpired that there are many other ancestral haplotypes, such as 57.1 (A1, B57, DR3) which must have been conserved since before the Out-of-Africa migration i.e., during thousands of generations and meioses (Kay et al., 1988). There were other interesting examples such as 7.1 (A3, B7, DR15) which contrasted with 8.1 in that it is protective with respect to Insulin Dependent (or Juvenile) Diabetes Mellitus (Degli-Esposti et al., 1992). How could this be explained?

By the 1990s, it was obvious that these ancestral haplotypes did more functionally than simply carrying HLA class I and II alleles. For example, 8.1 affects the concentrations of TNF and IgA in the plasma and the development of hyperplasia within the thymus (French and Dawkins, 1990; Vandiedonck et al., 2004). So, there are still many fascinating questions to be resolved. How do these conserved sequences do what they do?

Some of these questions are being addressed by Okano et al. (2020), Kulski et al. (2021a), Kulski et al. (2021b), Cun et al. (2021), and Tay et al. (2021). These groups are discovering new mechanisms of gene regulation embedded within the non-coding regions of Ancestral Haplotypes. As proposed by Kulski, Retroviral like elements may have several roles. Firstly, they can generate sequences involved in *cis* and *trans* interactions. Secondly, they can introduce conserved polymorphism associated with duplication.

## Mechanisms

Fortunately, we now have the benefit of Miro Radman’s provocative proposal based on his pioneering work on the actual mechanisms involved in mutation and recombination (Radman, 2022). Perhaps polymorphism can be self-sustaining. “The mismatching of DNA sequences and the recognition of mismatched base pairs by mismatch repair (MMR) proteins are the determinants of .... conservation of highly polymorphic genetic motifs.” The sequence differences and the activity of the MMR prevent recombination which would otherwise fragment the CPS.

How much sequence difference is required in order to conserve polymorphism by preventing recombination? Radman concludes that a minimal figure is of the order of 1 difference for every 30 base pairs in bacteria and 1/200 bp in lower eukaryotes. In mammals, the figure may be 1/400 bp. Interestingly, earlier comparisons of MHC ancestral haplotypes revealed sequence diversity sufficient to prevent recombination according to Radman’s predictions (Gaudieri et al., 1997, 1999; Longman-Jacobsen et al., 2003; Lloyd et al., 2016). The careful study by Smith et al. (2006) was also consistent with the Radman number for mammals. In a landmark study (Traherne et al., 2006), cite 3.4 to 3.6 differences per 1,000 bp, suggesting that there may be a buffer or safety zone between 1/300 and 1/400. Further, it appears that a linear increase in sequence divergence leads to exponential decrease in homologous recombination. The figure illustrates how Radman’s predictions result in conservation of polymorphic haplotypes. Blocks are conserved because sequence differences prevent recombination; these haplotypes are inherited unchanged from distant ancestors. However, as shown in the figure, some reshuffling is possible when a homozygous block is present, with the implication that frequent CPS can diverge.

No doubt there are additional factors and species differences, but it does appear reasonable to conclude that a certain amount of polymorphism, *ipso facto*, results in conservation. Critical, however, is that this safety zone for conservation must be retained over generations.

## Implications

The implications must be profound and extend to other genomic regions in Humans and, presumably, other vertebrates. We see these CPSs as the keys to an understanding of the fate of different individuals within the species. They can provide a type of memory for recurring challenges. They allow a concept which complements survival of the fittest with survival of the experienced.

Thus, the availability of tried and tested CPS permits some optimism, at least with respect to recurring events whether infectious or environmental and, also, with respect to changes in food and behavior. There is less cause for optimism in the case of new man-made challenges, such as population growth and biological warfare, as just two examples.

The further implications of CPSs deserve attention and might include, *inter alia*:

(a) Heterozygous advantage.
(b) Shuffling within the monomorphic sequence (see Figure 1) so as to increase diversity.
(c) Caveats on the use and interpretation of studies of homozygous cell lines.
(d) Strategies for threatened populations.
(e) Selection of preferred traits in livestock.
(f) Self vs. non-self recognition and autoimmunity.

## Conclusion

Conservation of Polymorphic Sequences associated with gene duplication may achieve the Radman number and thereafter provide stability of inheritance of multigenic traits.
